# Health-related quality of life of children and adolescents with osteogenesis imperfecta: a cross-sectional study using PedsQL™

**DOI:** 10.1186/s12887-018-1077-z

**Published:** 2018-03-02

**Authors:** Ana Paula Vanz, Juliana van de Sande Lee, Bruna Pinheiro, Marina Zambrano, Evelise Brizola, Neusa Sicca da Rocha, Ida Vanessa D. Schwartz, Maria Marlene de Souza Pires, Têmis Maria Félix

**Affiliations:** 1 Child and Adolescent Health, Universidade Federal do Rio Grande do Sul, Porto Alegre, Brazil; 2 Division of Pediatric Endocrinology, Hospital Infantil Joana de Gusmão, Florianópolis, Brazil; 3 Medical Sciences, Universidade Federal de Santa Catarina, Florianópolis, Brazil; 4 Medical Sciences: Psychiatry, Universidade Federal do Rio Grande do Sul, Porto Alegre, Brazil; 5 Medical Sciences, Universidade Federal do Rio Grande do Sul, Porto Alegre, Brazil; 6 Department of Genetics, Universidade Federal do Rio Grande do Sul, Porto Alegre, Brazil; 7 Health Technology Assessment in Clinical Genetics Research Group, Hospital de Clínicas de Porto Alegre, Porto Alegre, Brazil; 8 Medical Genetics Service, Hospital de Clinicas de Porto Alegre, Porto Alegre, Brazil

**Keywords:** Osteogenesis imperfecta, Quality of life, Sickness impact profile, Child, Adolescent

## Abstract

**Background:**

Osteogenesis imperfecta (OI) is a disorder of bone formation leading to low mineral density and fractures. Children and adolescents with OI require periodic medical follow up, corrective surgery, drug therapy and physical therapy, as well as specific daily care practices. In addition, they have an increased incidence of fractures, which require immobilization and cause severe discomfort and short-term disability. This study evaluated the health-related quality of life of children and adolescents with OI in two reference centers for OI treatment in southern Brazil.

**Methods:**

In this prospective cross-sectional study, the Pediatric Quality of Life Inventory (PedsQL^TM^) was applied in two university-affiliated reference centers for OI treatment in southern Brazil. Children and adolescents aged ≥ 5 years with clinical diagnoses of OI were included. Clinical data and socioeconomic status was evaluated.

**Results:**

The sample consisted of 52 children and adolescents with OI (aged 5-17 years); 26 (50%) participants with type I OI, 13 (25%) type IV, 12 (23.1 %) type III, and 1 (1.9%) type V OI. Physical and social functioning domains differed significantly according to clinical presentation of OI with lowest scores in the severe type (OI type III). Pain seems to be the variable that is most associated with impact on the PedsQL domains.

**Conclusions:**

Overall, this study revealed differences in physical functioning and social functioning in relation to OI clinical presentation. These results reinforcing the importance of the clinical management of these patients with the aim of functional improvement and importance of pain control.

## Background

Osteogenesis imperfecta (OI) is a disorder of bone formation leading to low mineral density and fractures [1, 2]. The incidence of OI is approximately 1/10,000-20,000 births [1]. It is usually caused by pathogenic mutations in the genes involved in the production of type I collagen, the main building block of bone, leading to reduced collagen production or structural failure. Approximately 80-90% of mutations occur in the *COLIA1* and *COLIA2* genes, which encode the α1 and α2 subunits of type I collagen, respectively [3]. Recently, several molecular studies identified additional genes involved in the biosynthesis of collagen as causing OI. However, the classification of OI that remains in widespread use is based on clinical and radiological features and defines five distinct types (I-V) [4, 5].

Many individuals with genetic disorders struggle with a variety of conditions that accompany chronic disease. Individuals with OI require periodic medical follow up, corrective surgery, drug therapy and physiotherapy, as well as specific daily care practices. In addition, they have an increased incidence of fractures, seen more frequently in children and adolescents, which require immobilization and cause severe discomfort and short-term disability [6]. Thus, OI is assumed to have a major impact on patients’ quality of life (QoL). Measurement and analysis of health-related quality of life **(**HRQoL) in individuals with genetic disorders, particularly OI, are essential for the evaluation not only of treatment outcomes, but also of patients’ well-being. This intervention may facilitate the work of professionals by modifying factors that go beyond health outcomes, including environmental, psychosocial, and school-related aspects, which may affect patients’ QoL. This study aimed to assess and characterize the HRQoL of children and adolescents with different types of OI.

## Methods

Participants in this prospective cross-sectional study were recruited by convenience sampling between December 2013 and January 2015 at two university-affiliated reference centers for OI treatment in two southern Brazilian states: the Clinical Hospital of Porto Alegre in Porto Alegre and Joana Gusmão Hospital in Florianópolis. The study was approved by the research ethics committees of both institutions and was conducted in accordance with the provisions of the Declaration of Helsinki. All parents or guardians provided written informed consent prior to patients’ inclusion in the study.

Children and adolescents were invited to participate after routine outpatient visits. No patient presenting for a first visit to the hospital was included in the study. The sample included children and adolescents aged ≥ 5 years with clinical diagnoses of OI type I, III, IV, or V. The OI classification adopted in this study was based on the clinical criteria established by Van Dijk and Sillence [5]. Patients diagnosed with and/or treated for anxiety and depression were excluded from the study.

Participants’ socioeconomic status was assessed using a validated Brazilian questionnaire based on the Brazilian Association of Research Companies Economic Classification Criterion [7]. This questionnaire yields a score that can be used to stratify the population into socioeconomic status ranges (A1, A2, B1, B2, C1, C2, D, and E), with “A” corresponding to the highest score and “E” to the lowest score [7].

Bleck’s criteria [8], modified by Land et al [9], were used to evaluate mobility: (0) not walking, (1) therapeutic walking, (2) household walking with or without assistance, (3) neighborhood or community walking with or without assistance, and (4) independently walking. For statistical calculations, classifications of 0, 1, and 2 were grouped and compared with classifications of 3 and 4. Two researchers (JVL and APV) collected clinical data.

Given the wide range of patient age at the time of evaluation, we calculated the rate of fracture according to the number of fractures/year. Patients were also asked whether they remembered having felt pain in the last month. Bisphosphonate treatment was investigated in patients receiving intravenous pamidronate (which is usually indicated for the more severe forms of OI with bone quality compromise), alendronate, or no medication.

HRQoL was evaluated using the Pediatric Quality of Life Inventory (PedsQL™ 4.0 Generic Core)*,* a generic assessment instrument that has been validated in Brazilian Portuguese [10]. This self-report instrument consists of 23 items in four domains: physical functioning (8 items), emotional functioning (5 items), social functioning (5 items), and school functioning (5 items). Responses are used to calculate total, psychosocial health, and physical summary scores. Scores were summarized according to a statistical model established by the authors of PedsQL™ 4.0 [11–16]. They were expressed as transformed scores on a 0-100 scale, with higher scores indicating better QoL. Children and adolescents filled out this self-report questionnaire. For children aged 5 to 7 years all questions were read by the researchers and answers were given according to a visual analogic scale as suggested by PedsQL™.

Continuous data were described by mean and standard deviation. Categorical data were presented as counts and percentages. Asymmetrical distribution variables were described by median, interquartile range, and range. Since the PedsQL score followed a nearly symmetrical distribution, mean groups of OI clinical presentation were compared using analysis of variance (ANOVA) with robust standard errors approach when required. Accordingly, the Tukey's and Dunnet's T3 (robust) tests were used for post hoc comparisons. Correlations between continuous symmetrical variables were computed using Pearson's product moment correlation coefficient or Spearman's rank correlation coefficient (rho) for situations where ordinal or not asymmetrical distributed variables we involved. A multiple linear regression model was used to simultaneously evaluate the impact of mobility, medical treatment, annual fracture rate, and pain score on the total PedsQL score and all the other component domains. The selection of these variables was more based on the underlying conceptual framework found in the specialized literature rather than on pure statistical significance. Asymmetrically distributed variables were log transformed before inclusion in the model. The standardized beta coefficient was used to asses and compare the magnitude of association of these factors with the different PedsQL domains in the multiple linear regression model. Statistical significance was set at p<0.05. Data were processed and analyzed using R version 3.3.0 and SPSS version 22.0.

## Results

The sample was composed of 52 children and adolescents with OI: 12 (23.1%) from the Florianopolis center and 40 (76.9%) from the Porto Alegre center. Patient characteristics are summarized in Table 1. Twenty-six (50%) participants had type I OI, 12 (23.1%) had type III, 13 (25%) had type IV, and one (1.9%) participant had type V OI. Twenty-five (48.1%) patients used pamidronate and one (1.9%) used alendronate; 19 (36.5%) patients were in physiotherapy. Thirteen (25%) patients used a wheelchair and 38 (73.01%) walked with support or independently. One patient was not included in the sample because he had a diagnosis of hyperactivity.

**Table 1:** Characteristics of study participants

Variable *n*= 52	Frequency no. (%)
Male	29 (55.7)
Age (years)	
5-7	13 (25.0)
8-12	24 (46.2)
13-18	15 (28.8)
Mean (SD)	11.2 ± 4.2
OI type	
I	26 (50.0)
III	12 (23.1)
IV	13 (25.0)
V	1 (1.9)
Treatment	
Sodium pamidronate	25 (48.1)
Sodium alendronate	01 (01.9)
Physiotherapy	19 (36.5)
Mobility^a^	
Wheelchair	13 (25.0)
Walk or walk with support	38 (73.0)
Pain in the last month (n=44)	
Yes	19 (43.2)
No	25 (56.8)
Annual fracture rate^b^	
Median (IQR)	0.91 (0.47 to 1.67)
Minimum to Maximum	0.06 to 6.33
Social assistance (n=42)	
Yes	20 (47.6)
No	22 (52.4)
Socioeconomic status (n=47)	
A	1 (2.1)
B1	3 (6.4)
B2	13 (27.7)
C1	13 (27.7)
C2	9(19.1)
D-E	8 (17.0)

^a^Bleck’s criteria [8], modified by Land et al [9], were used to evaluate mobility walking. For statistical calculations, classifications of 0, 1, and 2 were grouped and compared with classifications of 3 and 4. ^b^Number of fractures/year.

Thirty-seven (71.2%) children and 15 (28.8%) adolescents completed the PedsQL^TM^. In the total sample, mean physical functioning scores were lowest and mean social functioning scores were highest. In the analysis of mean domain scores according to clinical presentation, the physical functioning and social functioning scores differed significantly between participants with mild, moderate and severe forms of OI (Table 2).

**Table 2:** PedsQL^TM^ scores according to OI Clinical Presentation

PedsQL^TM^ score	Total sample	Clinical presentation*			
		Mild	Moderate	Severe	
	n = 52	*n* = 26	*n* = 14	*n=12*	*p* ^**^
Total	69.7 ± 13.1	71.3 ± 12.4	71.0 ± 13.2	64.8 ± 14.8	0.349
Physical functioning	61.9 ± 21.1	69.2 ± 17.2^a^	58.5 ± 23.7^a^	50.0 ± 14.8^b^	0.023
Psychosocial health	72.3 ± 12.7	71.9 ± 12.0	75.1 ± 11.7	69.7± 15.7	0.558
Emotional Functioning	66.3 ± 18.1	65.9 ± 16.3	67.0 ± 18.4	66.2 ± 22.9	0.984
Social functioning	77.7 ± 16.3	76.1 ± 19.3^a,b^	85.7 ± 8.0^a^	71.6 ±13.3^b^	0.007
School functioning	73.2 ± 15.2	74.5 ± 10.9	72.6 ± 17.8	71.2 ± 20.2	0.829

Data are presented as means ± standard deviations. *Clinical presentation- Mild (OI type I); Moderate (OI type IV and V); Severe (OI type III). **ANOVA, different index letters represent statiscally significant difference at the post hoc test

Clinical presentation was moderately correlated with physical functioning domain (*rho* = -0.38, *p* = 0.006) and socioeconomic status was inversely correlated with school functioning domain (*rho* = -0.31, *p* = 0.036). Age presented a weak correlation with the social functioning domain score, although significant (*r* = 0.29, *p* = 0.037). The annual fracture rate showed no significant correlation with any domain score.

Multiple linear regression analysis showed that pain score was statistically associated with all PedsQL domains except schooling. The pain score presented higher magnitudes of association with the total score, followed by psychosocial health, emotional, social, and physical functioning (Table 3).

**Table 3:** The impacts of selected patient characteristics on PedsQL^TM^ scores, as determined by multiple linear regression

PedsQL^TM^ domain/characteristic	Standardized beta coefficient	*p*
Total		
Mobility	0.22	0.19
Medical treatment	0,28	0.19
Annual fracture rate	-0.28	0.12
Pain score	-0.37	0.009
Physical functioning		
Mobility	0.35	0.03
Medical treatment	0,08	0.67
Annual fracture rate	-0.27	0.34
Pain score	-0.27	0.05
Psychosocial health		
Mobility	0.11	0.52
Medical treatment	0.33	0.12
Annual fracture rate	-0.23	0.20
Pain score	-0.36	0.012
Emotional functioning		
Mobility	0.10	0.56
Medical treatment	0.26	0.23
Annual fracture rate	-0.06	0.72
Pain score	-0.35	0.015
Social functioning		
Mobility	0.62	0.72
Medical treatment	0.20	0.35
Annual fracture rate	-0.20	0.28
Pain score	-0.28	0.015
School functioning		
Mobility	0.09	0.60
Medical treatment	0.27	0.23
Annual fracture rate	-0.27	0.17
Pain score	-0.20	0.18

Ninety-five percent confidence intervals were calculated and the level of significance was set at *p* < 0.05.

## Discussion

This study examined self-reported HRQoL of children and adolescents with OI using measures applicable to the age range investigated. Curiously, QoL is not frequently assessed in individuals with genetic disorders. A systematic review published in 2010 [17] identified 58 studies in which QoL was assessed using validated questionnaires in patients with genetic conditions. Only one of these studies examined patients with OI; QoL was assessed in an adult cohort using the SF-36, a validated health self-assessment questionnaire [18]. In a recently published scoping review that addressed the impacts of three genetic musculoskeletal diseases, 15 papers addressing OI were identified, but none focused on children and adolescents or assessed HRQoL with an available and validated inventory [19]. All articles used the term QoL, but the studies evaluated only physical factors such as functionality, and none used a tool designed to measure the QoL construct in children and adolescents.

Parents were formerly believed to be the appropriate informants about their children’s HRQoL. The current perspective is that children are able to speak for themselves, enabling researchers to avoid relying solely on parents’ opinions, which are influenced by their own experience and backgrounds [20–22]. Children and adolescents with chronic diseases requiring regular healthcare acquire knowledge about their health and are able to express their needs related to the disease. Often, their opinions about their health status are much more accurate than those of their parents or caregivers [23]. In this study, for example, two children using wheelchairs responded “never” to the item “It is hard for me to walk more than one block”; that is, their perception of “walking” was appropriate.

As reported by Fano et al [24], we found that patients with moderate and severe OI had lower physical functioning domain than did those with mild OI. This finding shows that the physical health component of QoL is compromised in severely affected patients, making them somewhat “uncomfortable.” In a qualitative study of children and adolescents with OI, physical health status was associated with the following themes: being safe and careful, reduced function, pain, fear, isolation, and independence [22]. In another study in which QoL related to olpadronate use was evaluated using the Self-Perception Profile for Children, the athletic performance score was lowest [25]. In the assessment of HRQoL using PedsQL^TM^ in patients with Duchenne muscular dystrophy, the most common inherited pediatric neuromuscular disorder and a cause of significant physical limitation, the physical domain score was lowest in children and adolescents [26].

Multiple linear regression analysis showed that pain affected total, physical functioning, emotional functioning, and psychosocial health scores. In OI, bone fragility results in recurrent fractures, leading to bone pain. Bone deformity also contributes to physical discomfort. A previous analysis of emergency department records demonstrated the need for additional effort and resources to address the under treatment of pain in children and adults with fractures in the emergency setting [27]. The authors stated that special attention should be given to analgesia for the very old and very young. They concluded that the education of providers on nonverbal options for measuring pain, especially in young children, may improve the measurement and documentation of pain status and facilitate recognition and treatment of pain in these vulnerable populations [27].

Socioeconomic status was correlated negatively with school functioning in the present sample, in which 26/52 (50%) patients’ family status was classified as B2 or C1. The 2012 Brazilian Institute of Statistics and Public Opinion survey showed that 29% of the Brazilian population belongs to class C1, followed by classes C2 (24%) and B2 (21%). Since 2005, improvement in the population’s economic status has been observed [28, 29]. In contrast to the findings of the present study, economic level was not correlated with QoL scores in our previous study of caregivers of individuals with OI [30]. In another study conducted in southern Brazil, socioeconomic level was correlated positively with environmental and social domain scores [31]. The differences can be explained by a small sample size and asymmetric distribution of the socioeconomic status in our study.

Age was positively correlated with the social functioning domain score in the present study. Although the literature does not contain reports of the impact of fractures on the QoL of children and adolescents, fracture is an important modifier of physical condition. It was one the six main themes identified in a qualitative interview-based study involving children and adolescents with OI, as well as their parents [22].

Seikaly et al [32], evaluated the impact of alendronate therapy on QoL in children with OI; however, no specific instrument was used to measure QoL. Their study focused on assessing individuals’ self-care and functional ability through application of a visual analog scale for pain and assessment of the number of days without pain. They found significant improvement in subjective well-being, pain, and analgesia use scores in subjects treated with alendronate. In the assessment of HRQoL using PedsQL^TM^, Fano et al [24] also identified pharmacological treatment as a modifying factor in a linear regression analysis. The design of the present study did not enable the comparison of treatment regimens; randomized studies are needed to characterize the main effects of drug therapy on OI patients' QoL.

This study has limitations. QoL is a construct with multiple definitions and it is based on qualitative variables, therefore, assessment using generic instruments that produce only quantitative variable values may have limited the analysis in this study. We believe that QoL can be assessed more accurately when quantitative and qualitative tools are used in combination. Moreover, the use of specific questionnaires is ideal for measurement of the impact of chronic disease on QoL with greater accuracy and sensitivity. To date, no specific questionnaire has been validated for patients with OI. Furthermore, the small sample size and convenience sampling limited the power this study. It should be noted that this is a usual finding when studying rare diseases and efforts were made to increase the sample size, recruiting subjects from different centers.

## Conclusions

Most chronic illnesses should be seen as stressors that may ultimately affect child development and can restrict children’s school and social performance, even in the family context. Estimation of the impact of chronic disease on the lives of children and adolescents is of fundamental importance for the development of interventions that can compensate for these deficits.

The measurement of QoL is gaining momentum and its consideration is having positive effects in health practice, as it allows the development and implementation of comprehensive interventions, rather than focusing only on the health/disease process. Overall, this study revealed differences in physical functioning in relation to OI severity and mobility, reinforcing the importance of the clinical management of these patients with the aim of functional improvement. Further studies in the same population with specific instruments for the measurement of QoL are required to support these results and provide more representative data.

## Abbreviations

CAAE: Certificado de Apresentação para Apreciação Ética
HRQoL: Health-related quality of life
OI: Osteogenesis imperfecta
PedsQL^TM^: Pediatric Quality of Life Inventory
QoL: Quality of life
